# An Operant Conditioning Method for Studying Auditory Behaviors in Marmoset Monkeys

**DOI:** 10.1371/journal.pone.0047895

**Published:** 2012-10-24

**Authors:** Evan D. Remington, Michael S. Osmanski, Xiaoqin Wang

**Affiliations:** Department of Biomedical Engineering, The Johns Hopkins University School of Medicine, Baltimore, Maryland, United States of America; Baycrest Hospital, Canada

## Abstract

The common marmoset (*Callithrix jacchus*) is a small New World primate that has increasingly been used as a non-human model in the fields of sensory, motor, and cognitive neuroscience. However, little knowledge exists regarding behavioral methods in this species. Developing an understanding of the neural basis of perception and cognition in an animal model requires measurement of both brain activity and behavior. Here we describe an operant conditioning behavioral training method developed to allow controlled psychoacoustic measurements in marmosets. We demonstrate that marmosets can be trained to consistently perform a Go/No-Go auditory task in which a subject licks at a feeding tube when it detects a sound. Correct responses result in delivery of a food reward. Crucially, this operant conditioning task generates little body movement and is well suited for pairing behavior with single-unit electrophysiology. Successful implementation of an operant conditioning behavior opens the door to a wide range of new studies in the field of auditory neuroscience using the marmoset as a model system.

## Introduction

The common marmoset (*Callithrix jacchus*) is an attractive model system for studying auditory processing and vocal communication due to its easily accessible auditory cortex and its high vocal activity in captivity [1]. This species has been used in recent years to study coding of pitch and complex spectral features in auditory cortex [2]–[5], temporal processing in auditory cortex [6]–[8], thalamus [9], and inferior collicullus [10], coding at different sound intensities [11]–[13], auditory cortex connectivity [14]–[17], auditory feedback mechanisms [18], and processing and control of conspecific communication in prefrontal cortex [19]. The marmoset has also recently become the first primate species in which germline expression of a transgenic modification has been achieved [20], broadening its potential as a model for cognitive function in disease.

Ultimately, developing an understanding of the neural basis of perception and cognition requires the ability to link brain activity with behavior. Our laboratory has developed techniques to study natural vocal behaviors of marmosets in free moving conditions [21]–[23]. However, answering questions regarding the neural basis of auditory perception often requires strict control of experimental conditions (for example, tests of spatial acuity demand a controlled head position) which is difficult to achieve in natural behavior conditions. Many animal models have well defined auditory behaviors for use in auditory physiology studies (e.g. ferret [24], [25] macaque [26], [27], cat [28], and rat [29]), as do many other species for behavioral studies (e.g. horses [30], gerbils [31], pigs [32], cows and goats [33]).

Previously, a conditioned avoidance task was used to measure absolute hearing thresholds in marmosets [34]. There have also been a number of studies using operant conditioning behaviors to study visual cognition in marmosets [35], [36]. We have developed an auditory operant conditioning task for the common marmoset. Subjects must lick at a feeding tube (equipped with an infrared photo-beam) during target sound presentation in order to receive a food reward while withholding licking when a target sound is not being presented. Most animals learned this behavior quickly and behaved consistently for relatively long periods of time. The task has already been employed in the measurement of a marmoset audiogram [37]. Here we present a complete description of the task and training procedures, additional considerations for marmoset training and behavior performance, and learning curves for 5 marmosets trained on this task. Crucially, we also show that this task can be coupled with single-unit electrophysiology recording without causing significant interference to the recording stability. We show examples from an animal performing a sound location discrimination task while single-unit recordings were conducted.

## Materials and Methods

### Ethics Statement

All experimental procedures were approved by the Johns Hopkins University Animal Care and Use Committee (protocol # PR09M469) and were in compliance with the guidelines of the National Institutes of Health. All surgery was performed under isoflurane anesthesia, and all efforts were made to minimize suffering.

### Animals and Housing

Marmosets were housed in individual cages in a large colony at The Johns Hopkins University School of Medicine. All animals were maintained at approximately 90% of their free-feeding weight on a diet consisting of a combination of monkey chow, fruit and yogurt and had ad libitum access to water. Subjects were tested once a day, five days per week between the hours of 0900 and 1800. During training and testing, animals were monitored by closed circuit infrared camera.

### Sound Delivery

Acoustic stimuli were generated digitally in Matlab (The Mathworks Inc., Natick, MA), loaded into a custom programmed RX6 multifunction processor (Tucker Davis Technologies, Gainesville, FL) and delivered by one or more free-field speakers located 1 m directly in front of the subject. All sound stimuli were generated at a 100 kHz sampling rate and low-pass filtered at 50 kHz. All behavior testing was carried out in single and double-wall sound attenuating chambers (IAC, Bronx, NY).

### Behavior Apparatus

The operant behavior setup includes a restraining chair, a behavior response apparatus, a reward delivery system, and a stimulus delivery and behavior control system. The restraining chair, designed for single neuron recording studies [38], allows a marmoset to sit in a comfortable and upright position and consists of a tube, a neck plate, and a foot platform. The tube and neck plate can be made from plastic or fashioned from steel mesh. Marmosets make behavior responses by licking at a feeding tube; responses are measured by a custom built lick detector which registers whether an infrared beam in front of the animal’s mouth has been interrupted. If the animal’s head is not restrained, this can also be accomplished by moving its face into the detector. A programmable syringe pump (NE-500, New Era Pump Systems, Wantagh, NY) delivers food reward through a disposable IV extension and into a custom machined lexan tube which can be positioned via a custom machined bracket fastened to the neck plate. For reward, we use a mixture of single-grain rice cereal (Gerber), strawberry and/or banana-flavor (Nesquik), a protein powder supplement (Nutiva), and baby formula (Similac). This mixture is nutritionally substantial and of relatively low viscosity for pumping efficiency; a single reward is between 0.1 and 0.2 ml and can be delivered within a few seconds. The speed of delivery is limited by mixture viscosity and pump speed and power.

**Figure 1:**
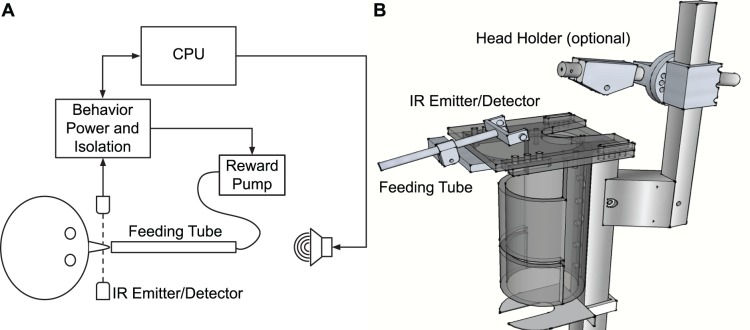
Marmoset chair and behavior setup. A. Marmoset chair with feeding tube, infrared lick detector, and optional head restraint mechanism for single-unit recording. The neck plate slides out to allow a marmoset to enter the chair from below. After securing neck plate, the feeding tube can be adjusted to create a comfortable reach for each monkey. B. Schematic of task setup. Sounds are played from free field speakers while marmosets lick to target sounds for a reward which is delivered by a syringe pump via a feeding tube. Lick responses are recorded when the infrared beam is broken by the animal’s face or tongue. Behavior apparatus are controlled by a personal computer and powered by a custom built power supply and electrical isolation module.

A computer running custom software written in Matlab and a custom programmed RX6 multifunction processor control behavior: Matlab software controls stimulus generation and behavior flow, while the RX6 unit serves to synchronize stimulus delivery, reward delivery, behavior responses and single-unit electrophysiology data (when applicable). A custom built power/relay module powers and electrically isolates the computer from the equipment inside the experimental chamber. The marmoset chair and feeding tube, along with a system schematic, are illustrated in Figure 1.

### Electrophysiological Recordings

Details of electrophysiology recording procedures can be found in previous publications from our laboratory [38]. One marmoset was surgically implanted with a dental cement head cap under sterile conditions with the animal deeply anesthetized by isoflurane (0.5–2.0%, mixed with 50% oxygen and 50% nitrous oxide). Head posts were embedded in the head cap to allow the animal’s head to be immobilized during recording sessions. To access the auditory cortex, small craniotomies (1 or 1.1 mm in diameter) were made in the skull over the superior temporal gyrus to allow for penetration by electrodes (impedance 2–5 Mohm at 1 kHz, AM systems) mounted on a micromanipulator (Narishige) and advanced by a manual hydraulic microdrive (Trent Wells). Action potentials were detected on-line using a template based spike sorter (MSD; Alpha Omega Engineering) and continuously monitored by the experimenter while data recording progressed.

## Results

### Go/No-Go Task

**Figure 2:**
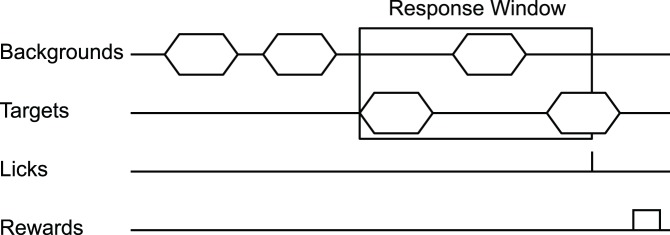
Behavior trial. After a variable number of background stimuli (or silent periods, for the detection task described here), targets begin alternating with the background stimuli/silent periods. If a lick is registered within the preset number of alternations, a food reward is given. After the animal has finished consuming the reward (as measured via the lick detector), the next inter-target interval begins with background stimuli or silent intervals. A lick outside of a target interval results in a timeout.

We chose to implement a Go/No-Go type task suited for measuring detection and discrimination thresholds. The task is similar to those previously described for non-human primates [39], [40]. Figure 2 illustrates the behavior paradigm. The objective in a Go/No-Go task is to respond to target sounds to receive reward while withholding responses when a target is not presented. Each behavior session is composed of a preset number of trials (typically 80–100), where each trial is composed of a variable duration “inter-target interval” and a fixed duration “response interval.” Inter-target interval duration is randomized between approximately 3 and 10 seconds but can be adjusted based on an animal’s behavior (see Response Shaping section below); the response interval is dependent on the number and duration of targets but is typically approximately 5 seconds in length. During an inter-target interval the subject hears either silence (in a detection task) or a series of background sounds (in a discrimination task). Behavioral responses during this time result in a mild punishment (see Response Shaping section below) and a restarting of the trial after the lick detector’s infrared beam is clear for a preset duration. After the waiting period ends, target stimuli are alternated with the background sounds during the response interval. The trial ends when the response interval has expired or a lick is detected during the response interval. Behavioral responses during this time are reinforced with approximately 0.1–0.2 ml food reward. During reward delivery, the software pauses to allow the subject to consume the reward, beginning the next inter-target interval the after the lick detector’s infrared beam is clear for a preset duration. If no response is detected, the next trial begins immediately.

A small percentage of trials are “catch trials,” which are identical in length to target trials in their timing and structure but in which no targets are delivered (i.e., only silence or background sounds are heard during the response interval). Thus, during a catch trial the response interval is indistinguishable from the inter-target interval from the animal’s perspective. A response during a catch trial response interval is referred to as a “false positive” (or false alarm). The false positive rate gives a measure of response specificity from which an experimenter can create an adjusted hit rate or calculate (along with hit rate) a measure known as d’ [41] in order to determine an animal’s perceptual sensitivity.

### Response Shaping

The procedure to train subjects to perform behavior tasks is referred to as response shaping. This process is controlled by custom software in conjunction with monitoring by the researcher. After an animal has been adapted to sit in the restraining chair and accept food through the feeding tube, training proceeds through two phases. *Phase 1*: food rewards are delivered following an auditory stimulus such as a white noise or pure tone while the animal’s behavior is monitored via closed circuit television and software. In this phase reward is not contingent on the subject’s behavior response. Animals soon start to associate the sound with food reward and begin showing anticipatory licking responses. *Phase 2*: reward delivery is made to be contingent on licking to the conditioning sound. The animal stays in phase two until the hit rate is consistently above 80% and the false positive rate is consistently lower than 25%. The animal is then considered trained, and testing on a detection task begins (for example, to determine hearing thresholds). Alternatively, animals can then be moved to a more complex discrimination task in which silent periods in the inter-target interval are replaced with audible background sounds. Where detection tasks are typically used to probe an animal’s hearing sensitivity, discrimination tasks are more generally used to test an animal’s ability to perceptually separate two sounds along some dimension.

Because the animal has been trained to lick in response to sounds, the presentation of audible background sounds during a discrimination task will usually bring a strong response from the subject at first. For this reason, it can be helpful to continue presentation of background sounds without pausing in response to licks until no licks have been recorded for several seconds, and then present the first target. Often, a monkey responds to this first target (provided it is easy to distinguish from the background) and continues to respond to further discrimination targets. Then, the process of false positive reduction repeats again until below the nominal level of 25%.

In some animals extra care is taken to reduce false positives. Any observer with some amount of internal noise will produce false positives, the probability of which is controlled by the response bias. In order to shift response bias and reduce false positives, several methods are employed, depending on each animal’s propensity to lick in error. One way to reduce false positives is to reduce the target probability [41], which can be achieved by increasing the inter-target interval length or the frequency of catch trials. Additionally, the number of targets below the perceptual threshold of the animal can be decreased. This doesn’t reduce stimulus probability *per se*, but rather reduces the number of targets for which a guess will result in a reward. For most animals, a ratio of response window length to inter-target interval length of about 0.5, less than 25% undetectable targets, and 20–30% sham trials is sufficient to keep false positives to an acceptable level.

For some animals, introducing an additional mild punishment for errors is helpful in reduction of guessing behavior, particularly early in training. We have used the following: (1) the inter-target interval is re-started and lengthened, (2) a “timeout” period (as described previously) is introduced, (3) the timeout is accompanied by a temporary shutting off of the chamber house light (blackout) and (4) the timeout period is accompanied by a puff of air delivered to the animal’s back or tail. For most animals, a timeout is sufficient to reduce false positives to acceptable levels.

**Figure 3:**
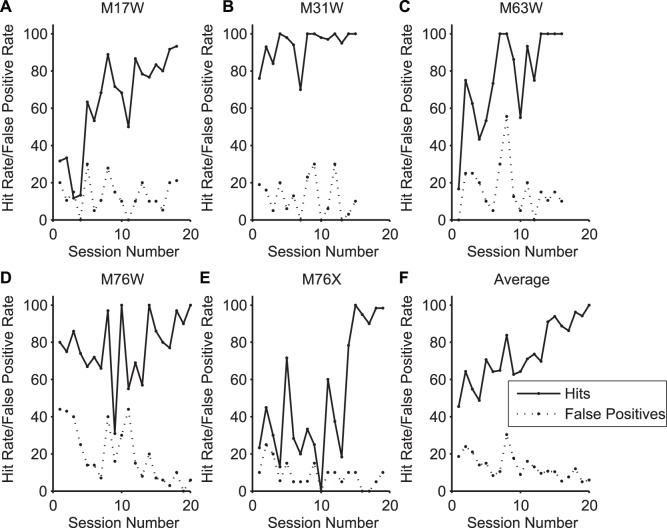
Learning curves. A–E. Learning curves for 5 naive marmosets performing an auditory detection task with broad band noise or pure tone stimuli. Data represent training *Phase 2* (see *Response Shaping*). Training is considered complete when 4 of 5 consecutive sessions have been completed with at least 80% hit rate and less than 25% false positives. Average time to train across all animals was 12 sessions with a standard deviation of 6 sessions. F. Average hit rate and false positive rate over all training sessions. Later sessions had fewer data points averaged due to some animals completing training more quickly than others.

### Performance in a Detection Task

To quantify task learning and performance, we trained five common marmosets (two male, three female) between two and five years of age on a Go/No-Go detection task. After marmosets became adapted to the restraining chair and first displayed anticipatory licking to sounds (*Phase 1, Response Shaping*), we quantified learning behavior through *Phase 2* of training. Hit rates increased and false positives decreased as the animal learned to associate sound with food reward, and training was considered complete when 4 of 5 consecutive sessions had been completed with at least 80% hit rate and less than 25% false positive rate. The “time to train” for a particular animal was the first session of *Phase 2* in which the subject reached this criterion of the 4 required. For 2 marmosets the detection sound was a 6 kHz pure tone, and for the other 3 the sound was a broad band noise token band-pass filtered between 2 and 32 kHz. These stimuli were chosen for the purposes of future psychophysical testing: the first group was later tested for pure tone detection thresholds [37], and the second group for spatial hearing acuity. Average time to train across all animals was 12 sessions with a standard deviation of 6 sessions. Figure 3 shows *Phase 2* learning curves for 5 animals trained over 2 to 3 weeks.

**Figure 4:**
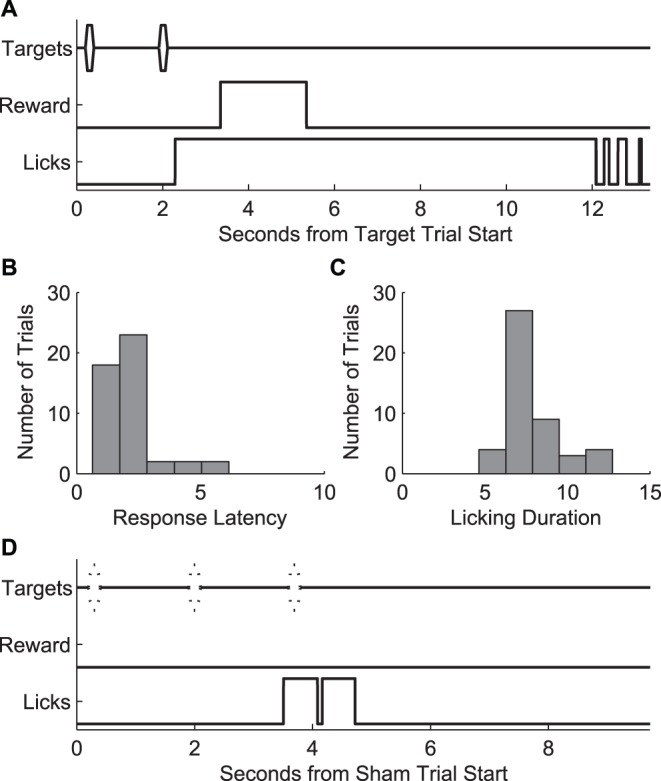
Licking behavior. A. Example of a licking response to a target trial along with reward and feeding behavior to target trials for a representative behavior session. B and C. Distribution of response latencies within the same session (B), measured as the elapsed time from the onset of the first target stimulus to the first lick, and lick durations (C), measured as the time from the first lick to the offset of the last lick. D. Example sham trial with an error response. Sessions consisted of 80 to 100 trials of which 30% were sham trials.

Figure 4 illustrates the time course of licking behavior and shows response latency and licking duration distributions for a representative behavior session. Response latency was measured as the elapsed time from the onset of the first target stimulus to the first lick. Licking duration was measured as the time from the first lick to the offset of the last lick. Sessions lasted 80 to 100 trials (30% of which were sham trials), after which we found a tendency for a reduction in motivation, likely due to animals becoming sated. The average session duration across all subjects at the end of the training period (last 5 sessions) was 32 minutes with a standard deviation of 6 minutes.

**Figure 5:**
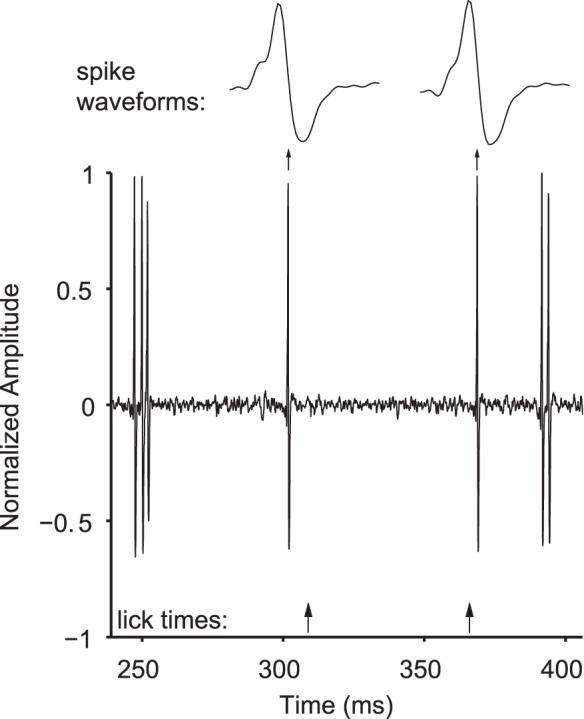
Single unit recording during behavior. Example of voltage signal, high pass filtered for spike sorting, from a high impedance microelectrode recording single unit activity in marmoset auditory cortex during task performance. Time is referenced to pre-stimulus delivery interval. The licking behavior can be performed without compromising recording stability (meaning that units can be held reliably) or signal quality. Note that spikes can be easily discerned both before and after a lick is detected.

### Application to Electrophysiology

A crucial goal of the behavior design was to allow the pairing of auditory perceptual tasks with single unit neurophysiology; we therefore designed the behavior setup specifically to be compatible with our neural recording methods. The setup (Figure 1) utilizes a modified version of the restraining chair used in our previous studies, allowing electrophysiology recordings to be performed as normal. However, it is important that licking, which results in jaw and tongue movement, does not adversely affect electrode stability or electrical signal strength. To show that single-unit recordings are possible during licking, we trained an implanted, head-fixed marmoset to discriminate sound source locations while recording single-unit responses during task performance. Although this task results in muscle movement of the jaw and tongue, as well as the presence of an electronic device in the vicinity of the recording equipment, we did not experience any obvious reduction in recording stability or electrical signal quality. Figure 5 shows a filtered voltage signal from an electrode recording single unit activity in marmoset auditory cortex during task performance. There is no appreciable movement or electrical artifact before or after lick detection, even though the animal’s jaw and tongue are active during these times.

## Discussion

### Comparison with Other Behavior Methods

Early marmoset psychoacoustic data was collected using negative reinforcement (shock avoidance [34]). Assuming that positive reinforcement would be more conducive to single unit recording stability, we tested several food-reward protocols. In addition to the licking strategy described here, we investigated both lever manipulation and eye position tracking. Behavioral reporting via lever movement seemed a logical choice, as it allows the reporting apparatus to be located far from the head and ears while potentially allowing for multiple response types (e.g. a left vs, right lever movement). Eye tracking has similar advantages: several saccade targets can be used, and equipment is out of the way, provided the high-speed camera can be positioned such that the acoustic field is not disturbed. There was some early success with eye tracking, but there was very little success with the lever. Lack of success with the lever task may have been due to the physically constraining marmoset chair. While experimenting with lever training, however, we found that marmosets were apt to lick at the feeding tube after a conditioning stimulus. In one telling case, a monkey which was being trained to pull on a manipulandum to obtain juice reward (not contingent on any target sound) never pulled on its own but very quickly began licking as soon as the manipulandum was moved by some external means. The tendency to lick to acquire food may be related to feeding patterns of marmosets in the wild, which include chewing holes in tree bark to feed on exudate [42]. Alternately, it could simply be that it is easier to train an action which is already necessary for food intake (marmosets must lick to ingest the reward regardless of whether reward delivery is contingent upon licking).

There are two potential disadvantages of lick reporting: first, the lick detector as described here has only one reporting option, ruling out a multiple forced choice task. This could be amended by adding a second feeding tube and lick detector, but would be more difficult in a head-fixed neural recording setup. Second, as the behavior apparatus is near the head and ears, possible acoustic field distortions should be considered. This issue would need to be addressed when conducting studies of spatial hearing, but it is possible to drastically reduce the amount of material holding the LED and phototransistor in place (for example by utilizing coiled wire). We believe that these drawbacks are far outweighed by the relative simplicity of training marmosets in the licking task.

### Conclusions

In this paper we have described an auditory operant behavior paradigm that is well suited to the study of acoustic perception in the marmoset monkey in which animals can be trained quickly. This paradigm takes advantage of the marmoset’s natural licking behavior. Thus far, it has been used to test absolute hearing thresholds in marmosets [37].

A promising feature of the behavior described here is its suitability for pairing with electrophysiological recording. The behavior measurement apparatus and reward delivery system were both designed to work in concert with current single unit recording procedures employed in the lab, and testing has shown that the setup is well suited for this endeavor (Figure 5). This creates the potential for achieving a more complete understanding of acoustic signal processing in the primate brain. Some of the most obvious applications for this task are the perception of vocal acoustics [18], [21] and pitch processing [3]. Another interesting question is how marmosets, a tropical arboreal species, perceive and process spatial sound information. Successful implementation of an auditory operant conditioning task adds to the existing attractiveness of the marmoset as a model for auditory processing and opens the door to new exciting discoveries in the field of auditory neuroscience.
